# “How PrEPared are you?”: Knowledge of and attitudes toward PrEP among overseas-born and newly arrived gay, bisexual, and other men who have sex with men in Australia

**DOI:** 10.3389/fpubh.2022.946771

**Published:** 2022-08-19

**Authors:** Budiadi Sudarto, Eric P. F. Chow, Nicholas Medland, Christopher K. Fairley, Edwina J. Wright, Jude Armishaw, Brian Price, Tiffany R. Phillips, Jason J. Ong

**Affiliations:** ^1^Central Clinical School, Faculty of Medicine, Nursing and Health Science, Monash University, Melbourne, VIC, Australia; ^2^Melbourne Sexual Health Centre, Alfred Health, Melbourne, VIC, Australia; ^3^Centre for Epidemiology and Biostatistics, Melbourne School of Population and Global Health, University of Melbourne, Melbourne, VIC, Australia; ^4^The Kirby Institute, University of New South Wales, Sydney, NSW, Australia; ^5^The Burnet Institute, Melbourne, VIC, Australia; ^6^The Peter Doherty Institute for Infection and Immunity, University of Melbourne and the Royal Melbourne Hospital, Melbourne, VIC, Australia; ^7^Department of Infectious Diseases, Alfred Health and Monash University, Melbourne, VIC, Australia

**Keywords:** PrEP, HIV, intersecting barriers, HIV prevention, overseas-born, newly arrived, GBMSM

## Abstract

**Introduction:**

Overseas-born and newly arrived gay and bisexual men and men who have sex with men (GBMSM) are at higher risk of acquiring HIV in comparison to Australian-born GBMSM. Pre-exposure prophylaxis (PrEP) is subsidized by the Australian government under Medicare, Australia's universal health insurance scheme, however many members of this population are Medicare-ineligible, which could prevent them from accessing PrEP. We wanted to explore participants' knowledge of and attitudes toward PrEP and their opinions of new PrEP modalities, namely injectable PrEP and PrEP implants.

**Methods:**

We conducted in-depth qualitative interviews between February 2021 to September 2021 with 22 overseas-born, newly arrived (<5 years in Australia) GBMSM of varying PrEP use. We asked their opinions of PrEP and their preferences of new PrEP modalities. Interviews were audio recorded and transcribed verbatim. We conducted a reflexive thematic analysis to interpret the data.

**Results:**

Participants' views reflect the intersections between systemic factors, such as Medicare ineligibility and the high cost of PrEP, with socio-cultural factors, such as lack of knowledge about PrEP, internalized stigma stemming from homo- and sex-negativity, and stigmatizing attitudes toward PrEP and PrEP users. For participants who were on PrEP, being community connected, having a positive relationship with doctors and nurses, and being informed of the option to purchase PrEP from overseas pharmacies at a low cost helped them to overcome some of these barriers. Additionally, there was a strong preference for injectable PrEP but not PrEP implants. Participants stressed the importance of providing a comprehensive information about PrEP specific to this population and to make PrEP free for all.

**Conclusions:**

We concluded that resources about PrEP specific to this population that address both systemic and socio-cultural factors are needed, and for these resources to be available in languages other than English. This is to coincide with on-going advocacy to increase the capacity of publicly funded sexual health clinics to provide multilingual PrEP services for people without Medicare, and to make PrEP free for all. These combined strategies have the potential to increase PrEP knowledge and uptake among this population.

## Introduction

HIV Pre-exposure Prophylaxis (PrEP) is a biomedical prevention strategy to reduce the risk of acquiring HIV and is particularly accessed by at-risk populations, including gay and bisexual men (cis and trans), and other men who have sex with men (GBMSM) (1–3). The current Australian Eighth National HIV Strategies include increasing PrEP uptake among at-risk populations, including GBMSM, as a means of further reducing the rate of HIV transmission in Australia, alongside increasing the rate of HIV testing and improving access to HIV medications for people living with HIV (4).

In Australia, PrEP was approved by the Australian Therapeutic Goods Administration in 2016. In 2018, PrEP was listed in the Pharmaceutical Benefits Scheme (PBS), allowing individuals with Medicare, Australia's universal health insurance scheme, to access PrEP at a reduced cost through their local pharmacies (1, 5, 6). The cost of generic brand PrEP under PBS is approximately AU$42 (equivalent to approximately US$30) per month, reducing to approximately AU$7 (approximately US$5) per month for concession card holders, including people on a low income, people with disability, and people receiving financial support from the Australian government (7). At the time of writing, the cost of unsubsidized PrEP for people without Medicare is around AU$45–160 (approximately US$32–117) per month from local pharmacies (8, 9). Individuals regardless of Medicare eligibility can import generic brand PrEP from overseas pharmacies from approximately AU$20 (US$15) per month (10).

Several Australian studies have shown a strong uptake of PrEP among GBMSM in Australia (2, 3, 11, 12), and the role of government subsidy to make PrEP affordable for individuals with Medicare that, in part, has contributed to an increase in PrEP uptake among GBMSM (13). However, there are still several factors that contribute to the hesitancy to take PrEP among some GBMSM. These include self-perception of not being at risk of acquiring HIV, concerns over the side-effects of PrEP and the general lack of awareness of PrEP as an HIV prevention strategy (1, 6, 11, 14). Among Australia's ethnic and migrant communities, additional intersecting factors that contribute to PrEP hesitancy include cultural taboos around sex and sexuality that play a part in the hesitancy to engage in HIV awareness and prevention, lack of awareness about PrEP due to lack of resources available in languages other than English, as well as unfamiliarity with the Australian healthcare system and stigmatizing attitudes toward PrEP users as sexually promiscuous therefore going against the cultural norm of monogamy (15–18).

Among ethnic and migrant GBMSM, including international students, studies have found that GBMSM participants were willing but did not use PrEP, in part because of the high cost of unsubsidized PrEP (1, 13, 18, 19). For international students specifically, limited hours permitted for paid employment up to 40 h per fortnight (20), and with the Australian minimum wage of around AU$20 per hour (or US$15 per hour) before tax (21), the hesitancy to take PrEP could intersect with income level and work restrictions. These may further intersect with Australian health policy that does not subsidize PrEP for individuals without Medicare (1, 19). Adding to this complexity, to date, only a handful of resources in Australia have been developed specifically for overseas-born GBMSM that involve community members and health practitioners from ethnic communities, such as Ending HIV's video in Chinese Mandarin that explains PrEP and challenges socio-cultural myths surrounding PrEP specific to Mandarin-speaking GBMSM (22). Moreover, given that PrEP is not readily available in all countries (23), including within the Asian Pacific region and countries such as China, India, and Malaysia (24), not all people coming to Australia are aware of PrEP as an HIV prevention strategy.

The intersections between systemic and socio-cultural factors that present as barriers to PrEP could play a part in the rise of new HIV diagnoses among overseas-born, newly arrived GBMSM in Australia. One study of HIV testing and new HIV diagnosis from Melbourne, Australia, between 2013 and 2017 showed that recent infection increased among newly arrived, Asian born GBMSM while falling in other groups (25). Additionally, in a study of people newly diagnosed with HIV in two Australian cities, Sydney and Melbourne, between 2014 to 2017, found that Asian-born international students made up more than half of new HIV diagnoses, and among all Asian-born GBMSM, <10% had access to Medicare, Australia's universal health insurance scheme (26). Likewise, a periodic report from the Australian national HIV surveillance data reported an increase of HIV infections among overseas-born GBMSM from 39.7% in 2016 to 49% in 2019 (27). Due to the COVID-19 pandemic, new HIV diagnoses among this population decreased to 44.8% in 2020 (27). This may be attributed to international border closures and reduction in sexual activities due to COVID19 lockdowns and social restrictions (28). Nevertheless, PrEP remains unsubsidized for people without Medicare, which could impact future HIV notification in Australia.

Based on past studies, epidemiological data and analysis, the importance of investigating the knowledge of and attitudes toward PrEP among this population became clear to us to understand some of the systemic and socio-cultural factors that can present as barriers to accessing PrEP in Australia. We were also curious to explore how individuals on PrEP or who had a history of taking PrEP overcame some of these barriers. Additionally, with recent development in new PrEP modalities, namely injectable PrEP and PrEP implants, we wanted to investigate participants' views of and preferences for these new modalities when they become available in Australia. A recent Australian study investigating GBMSM attitudes toward new PrEP modalities found that Long-Acting Injectable (LAI)-PrEP was preferred over daily oral PrEP, PrEP implants, and event-driven dosing (or PrEP on Demand) (29). However, this study did not differentiate between overseas-born and Australian born GBMSM.

Given the above available research and identified gaps in knowledge, this report aims to provide insights into the experiences of overseas-born, newly arrived GBMSM which could improve the promotion and advocacy of PrEP as an accessible HIV prevention strategy for this population.

## Materials and methods

We adopted a qualitative research methodology, and this is a novel approach given a limited number of available qualitative studies on PrEP in Australia. The study followed Consolidated Criteria for Reporting Qualitative Research (COREQ) guidelines to provide comprehensive reporting on qualitative research (30).

### Research team and reflexivity

The Research Officer, BS, conducted interviews with the participants. BS identifies as gay, non-binary, was born overseas, is a former international student, and is of Asian and Muslim backgrounds. BS has a Master's degree in Sociology focusing on sexuality and ethnicity. BS also has a professional background in HIV education and prevention. Additionally, they have been actively involved in the multicultural LGBTIQ+ (lesbian, gay, bi+, trans and gender diverse, intersex, queer, non-cisgender and non-heterosexual) communities for over 20 years. Due to BS' professional and community involvement, three participants recruited from BS' own network had prior knowledge of BS. This familiarity was used in the interview to add depth to the discussion. TRP, EPFC and JJO provided general supervision to BS. TRP assisted BS with the analysis. TRP is a research fellow in sexual health with experience in qualitative research in HIV and sexual health. TRP, EPFC, JJO and JA assisted with the recruitment. All authors contributed to the review of the journal article.

### Theoretical framework

BS used a theory-informed inductive approach in this research (31). Social determinants of health principles, an intersectional framework, and decolonizing research methodology were used throughout the research project. Social determinants of health principles are concerned with non-medical factors that influence health outcomes, including and not limited to health policies, social norms, and income which can have a direct influence on inequitable access to HIV care and prevention (32, 33). We complemented these principles with an intersectional lens to strengthen our analysis of the intersections between multiple systemic and socio-cultural factors contributing to health inequity. Kimberlé Crenshaw coined the term intersectionality in 1989 to explore the intersections of racism and sexism, and scholars and activists have since adopted it to investigate the intersections between multiple, marginalized identities caused by systemic inequality and injustice (34). An intersectional lens was used to explore health inequity experienced by GBMSM from ethnic minority communities (35, 36). The combinations of these two frameworks were suitable for this project as it allowed us to unpack various intersecting determinants that may contribute to participants' perception of and attitudes toward PrEP.

We also implemented a decolonizing approach to establish respectful, honest, and meaningful relationships between BS and the participants as equal contributors to the research project where their voices of strengths and resilience are used in initiating change (37). The framing of interviews as open and honest conversations based on shared cultural identities and lived experiences added depth to the data. BS' dual position as an insider and an outsider helped participants to feel a sense of connection while, at the same time, allowing BS to ask questions that were respectful of their cultural identities (38). Reciprocity is reflected whereby both parties benefited in the process (39); BS obtained the data needed for this study, while participants received information about PrEP and had a trustworthy contact person they could ask further questions after the interview. BS kept participants informed of the progress of the research to maintain trust and relationship.

### Participants and recruitment

In this study, participants were eligible if they:

- Were born outside Australia;
- Were aged 18 years or over;
- Identified as a gay or bisexual man (cis and trans), or a man who has sex with men;
- Had been living in Australia for <5 years;
- Had been sexually active with men in the past 12 months; and
- Had not been diagnosed with HIV.

Participants who had never taken PrEP, were not taking PrEP but had a history of taking PrEP, and who were taking PrEP were eligible for this study, as well as those with or without access to Medicare. We included participants who identified as non-binary (assigned male at birth and do not conform to the conventional gender binary of male and female) to reflect the gender diversity in the wider LGBTIQ+ communities (40, 41). Due to budget constraints, the interviews were only conducted in conversational English, thus requiring participants to have basic conversational English. Participants were selected using a purposive sampling method (42).

Various strategies were utilized in recruitment, including using digital posters on social media such as Facebook and Twitter, and recruitment emails sent to LGBTIQ+ and non-LGBTIQ+ community organizations requesting them to inform their members of this study. Nurses from the Alfred PrEPMe Clinic, a publicly funded clinic for individuals without Medicare to access PrEP, and health professionals from the Melbourne Sexual Health Center, the largest publicly funded STI/HIV clinic in Victoria, Australia, assisted with the recruitment by informing their clients of the study. A recruitment email was also sent to the researchers' professional HIV and sexual health networks, such as the Kirby Institute and the HIV/STI CALD and Overseas Born Gay Men Network. BS used their own personal network by posting a recruitment poster on their social media. A summary of places of recruitment and recruitment strategies can be found in Supplementary Table 1.

Potential participants contacted BS *via* emails, phone calls and text messages to indicate their interest. BS called the participants to introduce themself, confirm their eligibility, obtain demographic data, inform participants that the interviews were conducted in conversational English, and answer any questions relating to the study. In accordance with a critical aspect of decolonizing research methodology, BS allowed potential participants to ask questions about their own backgrounds relating to the project, including their professional backgrounds and lived experiences, to build trust and relationship before the interview (37). BS set a time to conduct the interview with eligible participants. BS emailed these participants with the Participant Information and Consent Form before the interview and obtained written informed consent. Recruitment was conducted between February and September 2021.

### Data collection and analysis

Interviews were conducted by BS over Zoom due to the COVID-19 health protocols and were audio-recorded using a digital recorder. BS used a semi-structured interview schedule to guide the conversation. Interviews were conducted in conversational English with minimal use of medical terminology. Participants received an information pack about PrEP after the interview was concluded. The PrEP information pack contained general information about PrEP, including ways to access and purchase PrEP without Medicare. Participants who were using PrEP or had a history of taking PrEP were also provided with the PrEP information pack to further enhance their understanding of PrEP. Additionally, participants received an AU$30 (approximately US$22) voucher by email to reimburse them for their time. Interviews were transcribed verbatim for data analysis. BS provided participants with a copy of their transcript so they could add, subtract, and edit information.

The sampling framework and interview topics are listed in Table 1. Information about new PrEP modalities was provided to the participants before we asked them of their opinions on injectable PrEP and PrEP implants. These are listed in Supplementary Table 2.

**Table 1:** Sampling framework and interview schedule topics.

**Sampling framework**
Gay and bisexual men (cis and trans) and other men who have sex with men
Newly arrived in Australia (<5 years)
Born overseas (outside of Australia)
HIV negative
Never on PrEP, not on PrEP but had a history of taking PrEP, and currently on PrEP
Medicare and no Medicare
**Revised during data collection**
More overseas-born, newly arrived GBMSM who were currently on PrEP
Able to communicate using conversational English
To include gender diverse individuals who were assigned male at birth and do not conform to the conventional gender binary of male and female, who have sex with men
**Eligibility criteria**
Born overseas
Living in Australia for <5 years
Aged 18 years or older
Able to communicate using basic English
Sexually active with other men (cis and trans) in the past 12 months
Gay and bisexual men (cis and trans) or a man (cis and trans) who have sex with men
**Interview schedule topics**
Knowledge of sexual health and PrEP in the participant's country of origin
Knowledge of sexual health and PrEP in Australia
Attitudes toward PrEP in Australia
**Revised during data collection to include additional questions**
Participants' opinions on new PrEP modalities (injectable PrEP and PrEP implants)

### Measures and analysis

BS used QSR International NVivo Plus software for data management, where interview transcripts were imported, collated, and read. BS then used Microsoft Excel to make notes of the themes that emerged from the data and copied and pasted interview excerpts from NVivo to Excel to record, manage, and revisit them during analysis. BS, JJO, TRP and EPFC met every 2 to 3 weeks to discuss the recruitment and data collection process, initial themes that emerged from the interviews, whether adjustments to the interview schedule and selection criteria were required, and preliminary data analysis to investigate whether any new or emerging themes were not previously identified that would change the results of the research. TRP assisted BS to read a selection of transcripts, confirmed coding and analysis, and investigated whether there were any discrepancies. TRP and BS reached a consensus that no new information provided by the participants would change the research results. The research team decided that data saturation was met after 22 interviews were conducted, and a decision was made to end the recruitment process.

BS conducted further analysis by using a reflexive thematic analysis framework. This framework recognized BS' positionality (both personal and professional) and assumptions as a researcher, and for BS to be reflexive in exploring the influence of their prior knowledge and assumptions on the research topic (43). BS immersed themselves in the data, coded the data, identified initial themes, and reviewed and refined the themes. BS then explored the connections between the themes and measured them against the research project's objectives, not as a linear process, but as ongoing and constantly evolving. The data were constantly analyzed to confirm or contradict pre-existing assumptions and find new meanings and knowledge.

## Results

### Participants' characteristics

In total, BS received 44 inquiries. Twenty-two people met the eligibility criteria and were interviewed. Participants who were interested but ineligible for the study included:

- Individuals who were not born overseas;
- People who had been living in Australia for more than 5 years;
- People who were living in Australia but were unable to return to Australia during the data collection period due to COVID19 and international border closures;
- People who did not respond to follow up communication to schedule an interview;
- Individuals who decided to opt-out from the research without providing a specific reason.

The average length of the interview was 58 min (range 41 to 118 min). Participants' characteristics are summarized in Table 2. Three major themes were pre-determined before the analysis derived from the research questions:

**Table 2:** Participants characteristics.

	***n* or mean [Range] (Percentage)**
Age	30 years [24–44 years]^a^
Length of time in Australia	2.5 years [1–4.5 years]^b^
**Place of Residence in Australia**	
Victoria	*16* (73%)
New South Wales	*3* (14%)
Queensland	*1* (4%)
Australian Capital Territory	*1* (4%)
Northern Territory	*1* (4%)
**Country of Origin**	
Southeast Asia (Malaysia, Singapore, Indonesia, and the Philippines)	*8* (36%)
East Asia (China)	*3* (14%)
South Asia (India and Nepal)	*3* (14%)
Middle East (Jordan)	*1* (4%)
Europe (the UK and the Netherlands)	*2* (9%)
North America (Canada)	*1* (4%)
South America (Brazil)	*4* (18%)
**Gender**	
Cis man	*20* (91%)
Non-binary	*2* (9%)
**Sexual Identity**	
Gay	*19* (86%)
Same-sex attracted	*1* (4%)
Pansexual	*1* (4%)
Demisexual	*1* (4%)
**Visa Status**	
Student visa	*13* (59%)
Working holiday visa	*2* (9%)
Work sponsored visa	*1* (4%)
Temporary visa	*2* (9%)
Bridging visa A	*2* (9%)
Permanent residence	*2* (9%)
**Medicare Eligibility**	
Eligible for Medicare	*4* (18%)
Not Eligible for Medicare	*18* (82%)
**PrEP Use**	
Never taken PrEP	*12* (54%)
Not currently taking PrEP but has previously taken PrEP.	*3* (14%)
Taking daily oral PrEP	*5* (22%)
Taking oral PrEP on demand	*2* (9%)
**Places of recruitment**	
University portal	*5* (23%)
Melbourne Sexual Health Centre	*2* (9%)
The Alfred PrEPMe clinic	*1* (4%)
LGBTIQA+ community organizations	*7* (31%)
Non-LGBTIQA+ community organizations	*3* (14%)
Professional network	*1* (4%)
Personal network	*3* (14%)

a *The standard deviation for the age of participants was 4.63*.

b *The standard deviation for the length of time in Australia was 0.89*.

- Did participants have some knowledge about PrEP in their country of origin? If yes, what were their experiences in understanding and accessing PrEP? If no, what were some of the barriers to understand and access PrEP?
- What are some of the barriers and enablers to understand and access PrEP in Australia?
- What are participants' opinions on new PrEP modalities (injectable PrEP and PrEP implants)?

The major themes that emerged from the data include:

- General lack of awareness of PrEP and access to PrEP in country of origin;
- Intersecting socio-cultural and systemic factors resulting in a general confusion about PrEP as an HIV prevention strategy, access to PrEP, and concerns over the cost of PrEP without Medicare;
- Intersecting enablers to understand and access PrEP without Medicare in Australia;
- General support for injectable PrEP but some concerns and hesitancy for PrEP implants.

The themes and sub-themes are presented in Table 3.

**Table 3:** Themes and sub-themes.

**Theme 1:**	**Theme 2:**	**Theme 3:**
Knowledge of and attitudes toward HIV and PrEP in the country of origin (Pre-determined)	Knowledge of and attitudes toward PrEP in Australia (Pre-determined)	Attitudes toward new PrEP modalities (Pre-determined)
1a. Socio-cultural homonegativity, HIV stigma and HIV knowledge (Emerged)	2a. Intersecting barriers in understanding PrEP and accessing PrEP without Medicare (Emerged)	3a. General positive attitude toward injectable PrEP (Emerged)
1b. Lack of access to PrEP in country of origin (Emerged)	2b. Overcoming barriers to understand and access PrEP in Australia (Emerged)	3b. General negative attitude toward PrEP implants (Emerged)

### Knowledge of and attitudes toward HIV and PrEP in country of origin

Several themes emerged from participants' opinions, and these are the impact of socio-cultural homonegativity and HIV stigma on HIV and PrEP awareness and knowledge, as well as lack of access to PrEP in their country of origin.

#### Socio-cultural homonegativity, HIV stigma, and HIV and PrEP knowledge

We noted the intersections between socio-cultural homonegativity and HIV stigma as influencing participants' knowledge of HIV and PrEP irrespective of their history of PrEP use. Internalized stigma stemming from sex- and homo-negativity was illustrated by the following participant:

“*It's really hard to be yourself in [my country]. The dominant people over there are religious, so it's really hard to think that I'm gay, I'm bi, I like men. It's really hard. We never talked about [sexual health]. No, it's taboo. We were never taught about STIs [sexually transmitted infections]. I knew about HIV, one of my friends got infected by HIV because she had casual relationships with many men. I think in school, there was one subject where we learnt about human and society, and HIV was mentioned, so I knew a little bit, but we never really talked about it. The first time I learnt about HIV, STIs and PrEP was at [a sexual health clinic in Melbourne, Australia].”*

*(PA015, 26 y.o., pansexual, cis man, Indonesia, 2 years in Australia, working holiday visa, never on PrEP)*

For another participant, even though PrEP could be accessed for free in Brazil, the cultural norm of monogamy as well as stigmatizing attitudes toward PrEP as an HIV prevention strategy for sexually active men may present as a barrier:

“*There are probably a lot of gay men who are not very open about their sexuality, so they're going to be a little bit scared of going to a sexual health clinic and get tested, being seen publicly as a man that has sex with men. People also stigmatize HIV. I think HIV is really, really attached to the gay community. When people think of HIV, they think of gay men. Also, growing up in a conservative country like Brazil, having a lot of sex is still a stigma, a taboo, in society. So, things that would go through my head are, “I shouldn't have that much sex, or I should only have a traditional way of relationship [monogamy]”. So, I think all of these things combined, and I think that's why, for a lot of people, [they don't like] the idea of taking PrEP, because it's assumed you want to have [lots of] sex.”*

*(PA002, 28 y.o., gay, cis man, Brazil, 1 year in Australia, international student, daily oral PrEP)*

Perceived homonegativity from medical practitioners in country of origin was reported by a participant as preventing him from discussing sexual health with his doctor:

“*[In my country], I used to do the [HIV] testing, you were telling the doctors that you were sexually active, but you're not going to discuss that you're homosexual, or you're sexually active with men, or whatever. It wasn't really easy. I didn't feel comfortable discussing about sexual health or discussing [sex] with them. We used to do our own homework, searching [information] about STIs [online].”*

*(PA008, 32 y.o., gay, cis man, Jordan, 1.5 years in Australia, temporary visa, daily oral PrEP)*

#### Lack of access to PrEP in country of origin

For many participants who were taking PrEP or had the experience of taking PrEP, the majority had heard of PrEP in their country of origin. However, they also said that information about and access to PrEP were not widely available. A participant's opinion below reflects the intersections between perceived homonegativity from his doctor, internalized stigma, and health policy that created a barrier for him to access PrEP in his country of origin:

“*[I went to] a government-owned clinic [in Sumatra]. The doctor was religious, and she was kind of judging me. I was feeling uncomfortable with the way she was asking me questions and I got the sense that she was doing it because she had to do it, it's part of her job, but she was not happy to be dealing with our kind of people [gay men]. I felt so down after that, I tried to suppress my curiosity [about HIV and STIs] and the desire to meet people. When I moved to Bali, I started [meeting people] again and I looked up online all of the STIs and all the sexual health and I heard about PrEP. But, I didn't pay too much attention to it back then because I wasn't sure how accessible it was in Bali, because [PrEP] was not subsidized by the [Indonesian] government.”*

*(PA014, 38 y.o., gay, cis man, Indonesia, 2 years in Australia, bridging visa A, PrEP on Demand)*

Participants from countries where homo- and sex-negativity were less prevalent than for participants from Asian, Middle Eastern, and South American countries also reflected on the difficulty they experienced accessing PrEP in their country of origin. A participant recalled that, in 2017, government health policy and hesitancy from some doctors to prescribe PrEP posed as a barrier for him to access PrEP in the United Kingdom (UK):

“*Accessing sexual health in the UK was much harder. The budget was much lower, and so, the system is under-resourced. Sometimes, it felt almost impossible to get care in the UK. I tried to get PrEP in the UK before I came [to Australia], but it was very hard, even if you could pay for it. Some doctors in the UK were very negative about it. Whereas, here, the system seemed to be more open about PrEP.”*

*(PA012, 44 y.o., gay, cis man, the United Kingdom, 4.5 years in Australia, permanent residence, PrEP on demand)*

### Knowledge of and attitudes toward PrEP in Australia

Participants, irrespective of their PrEP use, said they became aware of PrEP once they arrived in Australia through PrEP public health campaigns, university sexual health information sessions, dating apps for GBMSM, as well as sexual partners and/or peers. Despite these forms of information provision, participants also reflected some barriers in understanding PrEP, especially around accessing and purchasing PrEP without Medicare.

#### Intersecting barriers in understanding PrEP and accessing PrEP without Medicare

Many participants of varying PrEP use mentioned the systemic issue of PrEP not being subsidized for people without Medicare that resulted in the high cost of PrEP as being the main barrier to access PrEP. A few participants criticized Australian health policy for not subsidizing PrEP for people without Medicare:

“*I'm eligible to get a free [COVID] vaccine, because that's something the government recognizes as very important for your health; you do not need to be an Australian citizen [to get vaccinated] because it's [benefiting] the community, so [treat PrEP] in the same way; we are living in this community, so, make it economically cheaper so [people] like me, an international student, would take [PrEP].”*

*(PA007, 26 y.o., gay, cis man, India, 1 year in Australia, international student, never on PrEP)*

Minimum information specific to this population added to the confusion around access to PrEP without Medicare and whether their private health insurance would cover the cost of PrEP:

“*I think with PrEP intervention in this country, it's targeted at [financially] secure, White people. I think it's missing in the ethnic communities, especially international students. I think there is a lack of understanding [about PrEP], and the most challenging thing is not knowing what you have to pay and [whether PrEP] is covered by your [private overseas students health insurance]. There's a lot of lack of understanding in terms of where can I access it, is it free, how much do I have to pay; a lot of these [information] are completely missing. I think that is what is needed, being accessible with information [about PrEP].”*

*(PA013, 27 y.o., gay, cis man, India, 4 years in Australia, international student, daily oral PrEP)*

Indeed, many participants on a student visa expressed that they did not access PrEP because it was expensive, and this intersects with a systemic issue of the number of hours permitted to work and employment opportunities. As stated by a participant:

“*I would say, financially, right now, I got a casual job just last month, or two months ago, so there's some financial consideration, because I'm not on Medicare, being an international student, and from what I read, [PrEP] is costly. I don't know if I can afford it.”*

*(PA007, 26 y.o., gay, cis man, India, 1 year in Australia, international student, never on PrEP)*

Adding to this, several participants mentioned the lack of information about PrEP in languages other than English that could make it difficult for overseas-born, newly arrived GBMSM to understand PrEP, especially among those who are still learning the English language:

“*I know some [international students] come here to learn English, so you know, they're still learning English, and we know information about PrEP can be so confusing, it can be so medical.”*

*(PA002, 28 y.o., gay, cis man, Brazil, 1 year in Australia, international student, daily oral PrEP)*

Indeed, only one participant was able to recall seeing resources about PrEP in a language other than English:

“*Is there any information about PrEP [in Chinese]? I've seen it at [the doctor's] office, but apart from that, I've never seen it anywhere else.”*

*(PA022, 24 y.o., gay, cis man, China, 3 years in Australia, temporary graduate visa, daily oral PrEP)*

Minimum information not only resulted in confusion about the cost of PrEP without Medicare, but also the long-term and short-term side effects of PrEP, especially among participants who were never taking PrEP:

“*I have no idea about the side effects [or PrEP]. I think PrEP doesn't have any [side effects], but I'm still scared because everybody has a different body.”*

*(PA015, 26 y.o., pansexual, cis man, Indonesia, 2 years in Australia, working holiday visa, never on PrEP)*

These systemic factors intersect with socio-cultural factors and create further barriers to accessing PrEP. Several participants mentioned that homo- and sex-negativity in their country of origin could result in some individuals having a low knowledge of, and the reluctance to seek information about, HIV and PrEP, especially among newly arrived international students from Asia:

“*For us Asians, we don't really discuss [sex] in public, so when I first came here, I was a bit shy. For the first few months, I was a bit shy, because [sex] is not something that we talked about openly. I think a lot of international students are struggling with it. If they are not willing to reach out to find information, then they would not [do it] because there's this [cultural] barrier.”*

*(PA006, 29 y.o., gay, cis man, Malaysia, 2 years in Australia, international student, never on PrEP)*

A participant noted that moving to Australia could be seen as sexually liberating by some international students due to a more open attitude toward same-sex attraction than in their country of origin. However, they may not be aware of the risk of acquiring HIV due to limited knowledge about HIV and PrEP. This could further intersect with a systemic issue of not being eligible for Medicare and thus not being able to access government-subsidized PrEP:

“*People moved to other countries for many reasons: economic, cultural, but also for sexual reasons. Imagine if you live in a culture, a country, where gay sex is less available, and you come to Australia, and you think, ‘Oh, what an amazing sexual supermarket!' The problem with cultural change is that, if you don't know about HIV, you don't know about PrEP, you might not understand your risk [of acquiring HIV], and therefore might choose not to be on PrEP. Imagine people who are young, who are just starting to have sex, they may have some ignorance about HIV and PrEP. I'm also wondering whether international students have access to the Australia healthcare system [Medicare]. That might also be a barrier [to PrEP].”*

*(PA012, 44 y.o., gay, cis man, the United Kingdom, 4.5 years in Australia, permanent residence, PrEP on demand)*

For a few participants, being new to Australia also resulted in confusion about navigating the Australian healthcare systems to access PrEP:

“*One thing that I haven't yet understood, how exactly can I have access to PrEP being an international student living in Australia? How much would that cost? I've had that conversation with a health professional at the health clinic, but it wasn't very clear to me. Do I have to go to a [doctor], and then, will they [then] refer you to a sexual health clinic? I'm not sure. Where do I get [PrEP]? Do I go to a normal chemist to get it, or do I order it [online]? I get very confused with [the process].”*

*(PA009, 30 y.o., gay, cis man, Brazil, 2 years in Australia, international student, never on PrEP)*

Adding to the complexity, several participants mentioned stigmatizing attitudes toward PrEP and PrEP users as influencing their perception of PrEP. For a particular participant, the stigma came from the promotion of PrEP as an HIV prevention strategy for sexually active individuals:

“*From the information that they gave me at [a sexual health clinic], take [PrEP] every day if you're sexually active. They mentioned it a lot. So, when [someone] say that [they are] on PrEP, from my perspective, I would think, ‘Okay, you must be really sexually active, have a lot of different sexual partners'. So, I don't take [PrEP] because I'm not sexually active, and it's so expensive.”*

*(PA003,29 y.o., gay, non-binary, Malaysia, 2.5 years in Australia, bridging visa A, never on PrEP)*

A participant noted that some individuals from homo- and sex-negative ethnic communities in Australia may have internalized stigmatizing attitudes toward PrEP, seeing PrEP as symbolizing sexual promiscuity that goes against the cultural expectation of monogamy and the high moral standard associated with it:

“*PrEP in your bag means you're sleeping around, why would you have PrEP if you're not fucking around? Because they have sex with multiple partners, it's like, ‘Oh, disgusting!' or like, ‘You're not loyal, basically, you have no morals. You've come to Australia, you've changed, you've become like the Western man.' That's where a lot of this comes from, this whole internalized stigma; if you sleep with more than one person, you are a bad person, because it means that you can't keep your dick in your pants, you're super horny, you have no morals. I think for some people, they feel, ‘Oh my gosh, I'm changing so much as a person and that frightens me. I'm now turning into the person that I used to judge when I was younger. Is my younger self going to be ashamed of me? I promised I would not change too much, but here I am doing all these things.' And then you start feeling a lot of regret and disgust and so on and so forth. So, it's this intersection between internalized stigma and culture.”*

*(PA005, 32 y.o., demisexual, cis man, Singapore, 3.5 years in Australia, international student, never on PrEP)*

Internalized stigma may also result in the fear of being judged by medical practitioners, and a few participants felt that this could prevent some individuals from talking about sexual health and PrEP with their doctors:

“*I think there's a bit of stigma around [PrEP]. They feel like they would be judged by their doctor. I'm quite open about my [sexual practices], but I feel like, if you are someone that cares about what [your doctor] are thinking, then it would matter. If your [doctor] is not friendly, people might find it hard to talk about these things.”*

*(PA001, 29 y.o., gay, cis man, Malaysia, 2 years in Australia, international student, never on PrEP)*

We also noted that a few participants mentioned that their attitude toward PrEP has been influenced by their cultural upbringing around health and medicine. For these participants, cultural practices that prioritize traditional medicine and herbal remedies added to their concerns over the safety and side-effects of PrEP:

“*With any medication, there has to be some sort of side effect. I just have the philosophy where I don't want additional chemicals in my body. I think there is an element of truth where people from Asian cultures are maybe a bit more frightened taking medication and stuff like that. A lot of Asian medicine is based on more naturopathic sort of elements, or remedies, whereas a lot of Western medicine is more science, you know, chemistry based. So, I think about that, I don't think it's worth getting all the side effects [of PrEP].”*

*(PA016, 31 y.o., gay, cis man, Nepal, 2 years in Australia, work sponsored visa, never on PrEP)*

#### Overcoming barriers to understand and access PrEP in Australia

Participants who were on PrEP or had a history of using PrEP recalled some factors that helped them overcome some barriers. For a few participants, having peers, sexual and/or romantic partners who were knowledgeable about PrEP helped them to enhance their understanding of PrEP:

“*Before I came here, I had no knowledge about PrEP. I came here and then I made many friends and most of them were on PrEP, so it helped to get more information. I also remember a Facebook group, a famous one, PrEP Access Now, then I started to understand more about PrEP.”*

*(PA019, 35 y.o., gay, cis man, Brazil, 3 years in Australia, international student, not on PrEP with a history of taking PrEP)*.

Being socially connected also assisted these participants in finding information about ways to purchase PrEP without Medicare, including importing PrEP from overseas pharmacies:

“*The first time I bought PrEP at a pharmacy, the best price that I could find was AU$70 for one month. When I finished the bottle, I was thinking, ‘What am I going to do now, where am I going to buy this? Do I have to spend a lot of money, again?' Then, my boyfriend said that he used to order PrEP from overseas when PrEP was not part of PBS. So, I ordered from [an overseas pharmacy] and it's cheaper [than buying PrEP locally]. Cost was definitely a big issue. I don't know if I would keep taking PrEP if there was no option for me to pay less.”*

*(PA002, 28 y.o., gay, cis man, Brazil, 1 year in Australia, international student, daily oral PrEP)*

For other participants, their doctors and nurses helped them gain a better understanding of PrEP, including information about purchasing PrEP without Medicare. A participant was hesitant to discuss sexual health with his doctor in his country of origin due to perceived homonegativity. The ability to talk openly without judgement with his doctor in Australia helped him to understand PrEP and access to PrEP without Medicare:

“*I went to the university clinic, and I discussed with my [doctor] regarding my sexual orientation, and that I'm interested to take PrEP. I didn't know how effective this medicine is. For me, it was something new that I heard about, but I didn't know if it's working or not. She gave me an explanation how the medicine works, and how to take PrEP. I felt [that it was] a [safe] space to discuss my sexual health. She was very supportive; she was very informative. She told me everything about [PrEP], gave me the website where I can buy PrEP from [overseas] and at an affordable price.”*

*(PA008, 32 y.o., gay, cis man, Jordan, 1.5 years in Australia, temporary visa, daily oral PrEP)*

A few participants mentioned the role of publicly funded sexual health clinics to not only access free STI and HIV screening, but also to gain information about PrEP especially on the option to import PrEP from overseas pharmacies at a low cost:

“*I'm not a citizen, I wasn't sure if I would be able [to be on PrEP] if I'd have to pay full price for it. I guess, when it comes down to it, the cost of [PrEP] was the biggest thing [to consider] about taking PrEP. Then, I went to [a free sexual health clinic], I did my test, and the nurse gave me information about [an overseas online pharmacy] to buy PrEP and it was incredible, very affordable.”*

*(PA018, 33 y.o., gay, cis man, Canada, 2 years in Australia, working holiday visa, daily oral PrEP)*

One participant was involved in EPIC-NSW, a large PrEP implementation study in New South Wales, Australia, between 2016 and 2018, and it was through sexual health nurses involved in the study that he was able to access PrEP for free even after the study had finished:

“*I never paid for PrEP. I was part of a study [EPIC-NSW] so I didn't pay for it, and I kept [getting PrEP] without paying for a while, then they stopped the study, and I was going to stop taking PrEP because I didn't know how to pay for PrEP. I know [that] for some people, [the cost of PrEP] is not much, but for me, [the cost] would make a difference. I was like, ‘Okay, I will just stop taking it because I cannot pay for it'. But, my nurse found a way for me to still get [PrEP] from the hospital [for free] so I kept getting it. At the moment, I'm in a relationship so I [don't] take [PrEP].”*

*(PA011, 30 y.o., gay, cis man, Brazil, 3 years in Australia, international student, not on PrEP with a history of taking PrEP)*

Health professionals also played a role to ease participants' concern over the side-effects of PrEP, especially among participants who came from a culture that does not favor Western medicines. A participant commented that having a doctor from a similar cultural background helped him to overcome some of the cultural attitudes toward Western medicines and PrEP:

“*[At first], I didn't believe in PrEP, because my grandpa is quite traditional; he's a Chinese medicine doctor. He told me anything from Western medicine is not good for your kidneys. That's why I didn't [take] PrEP because I was concerned it would affect my kidneys and my bones. [This was] until my partner told me that it's fine, and it's a medication that's protecting you. Then I met my doctor at [a free sexual health clinic in Melbourne, Australia]. There's nothing that you feel you need to leave out. I'm so lucky [to have] met [my doctor]. He gave me information about PrEP. He told me it's a good medication to protect yourself [from HIV].”*

*(PA022, 24 y.o., gay, cis man, China, 3 years in Australia, temporary graduate visa, daily oral PrEP)*

### PrEP modalities: Interest in new ways of taking PrEP

Following a recent development in new PrEP modalities, we were interested in exploring participants' opinions on injectable PrEP and PrEP implants. During the interview, all participants received information about these new PrEP modalities, and two themes emerged from their opinions: a generally positive attitude toward injectable PrEP, but an overall negative attitude toward PrEP implants.

#### General positive attitude toward injectable PrEP

Most participants, irrespective of their PrEP use, expressed their interest in injectable PrEP, and they mentioned the convenience of an injection every 2 months as appealing:

“*The convenience [of it], because if it's just one shot every two months, then I can have a very secure, active sexual life, then why not? But if you're taking [PrEP pill] every day, that is a hassle.”*

*(PA003,29 y.o., gay, non-binary, Malaysia, 2.5 years in Australia, bridging visa A, never on PrEP)*

For a few participants who were already taking non-HIV related daily medications, injectable PrEP meant taking one less medication:

“*I already take a pill every day because of [a health condition], and it's annoying that you have to take a pill every day, and PrEP would be another one. So, if you just have an injection, then, why not?”*

*(PA011, 30 y.o., gay, cis man, Brazil, 3 years in Australia, international student, not on PrEP with a history of taking PrEP)*

For a particular participant, an injection was seen as less confronting and more discreet than PrEP pills, as he previously mentioned that a bottle of PrEP symbolizes an active sex life that contradicts his cultural upbringing and the norm of monogamy:

“*Not to have that bottle of pills just staring at me, I think that would be quite nice. You know, sometimes when you have friends over for [a meal], you don't want them to see [a bottle of PrEP].”*

*(PA005, 32 y.o., demisexual, cis man, Singapore, 3.5 years in Australia, international student, never on PrEP)*

One participant felt that injectable PrEP would positively contribute to the collective effort to reduce HIV transmission once it has been proven safe to use:

“*If it can be proven safe, no harm in the long run with your organs, then I would really much [be interested], it would really help [to reduce] the number of HIV [transmission]; at least you can do something to prevent the disease.”*

*(PA017, 32 y.o., same-sex attracted, non-binary, the Philippines, 2.5 years in Australia, international student, not on PrEP with a history of taking PrEP)*

Some participants however still expressed concerns over the cost of injectable PrEP without Medicare, as well as its potential side effects:

“*It depends on the cost, and then maybe any side effects that it may have. But as I understand it [right now], if it's easier to do [than daily oral PrEP], then why not?”*

*(PA016, 31 y.o., gay, cis man, Nepal, 2 years in Australia, work sponsored visa, never on PrEP)*

Not all participants were open to the idea of an injectable PrEP. One participant believed that it would be easier to remember taking daily oral PrEP than to get an injection every 2 months:

“*I would prefer to take it every day because I feel like taking an injection every two months, I would totally forget it, unless someone could send me a text message or something, but I'd still need to go to the [clinic]. Taking a pill, it would be much easier.”*

*(PA020, 26 y.o., gay, cis man, China, 3 years in Australia, international student, never on PrEP)*

A participant viewed the ability to purchase PrEP from the local pharmacy as more convenient than having to go to a clinic every two months for an injection:

“*I think I would prefer the pills because you can just go to the pharmacy and get some. It's easier, and more convenient, even though you have to take one [pill] every single day.”*

*(PA021, 35 y.o., gay, cis man, the Netherlands, international student, 2.5 years in Australia, never on PrEP*

Another participant could see the benefit of injectable PrEP but still preferred daily oral PrEP because he viewed taking a pill as symbolizing taking personal responsibility to prevent HIV:

“*It's a good option to have – it depends on person to person; some people would rather have a pill every single day to be safe and some people will be like, ‘Okay, yeah, one injection every two months is fine'. For me, taking PrEP every night with my dinner makes me feel like, ‘Okay, there's the renewal of my 24 h of safety'.”*

*(PA013, 27 y.o., gay, cis man, India, 4 years in Australia, international student, daily oral PrEP)*

#### General negative attitude toward PrEP implants

We found that PrEP implants were not favored by the majority of the participants irrespective of their experience using PrEP. Unfamiliarity with an implant and viewing it as a foreign object, as well as perceiving the process of inserting PrEP implants as intrusive and painful, contributed to their hesitancy:

“*A little bit scary to me if you just think about implants. The first thing came into my mind was, does it hurt? I would rather take the needle shot because from childhood, you have taken so many shots, but the implant is kind of a new thing to me.”*

*(PA010, 25 y.o., gay, cis man, China, international student, 2.5 years in Australia, never on PrEP)*

Another participant preferred a daily oral PrEP, injectable PrEP, or abstaining from sex altogether instead of taking PrEP implants:

“*That sounds terrifying, putting something in your body; it sounds interesting, but it's still terrifying. With implants, it's something in your body that is there all the time. I don't know, the idea, mentally, doesn't sound good. I prefer not to have sex over putting something in my body. Or just take PrEP daily, that's not really hard. It's just a pill, I take it every day, that's it.”*

*(PA008, 32 y.o., gay, cis man, Jordan, 1.5 years in Australia, temporary visa, daily oral PrEP)*

One participant, however, was interested in PrEP implants, citing the convenience of only needing to replace an implant once or twice a year as appealing:

“*I only have to worry about it twice a year. That's fine. It's not at the back of your mind as like taking [PrEP] every day. I'm taking an implant!”*

*(PA004, 26 y.o., gay, cis man, Malaysia, 4 years in Australia, permanent residence, never on PrEP)*

### Concluding statements: A case for free PrEP

In their concluding statements, many participants reiterated their opinions on the need to further inform overseas-born, newly arrived GBMSM of PrEP to increase PrEP uptake among this population. A participant reflected that PrEP still need to be widely understood by this population before providing them with various options to take PrEP:

“*I think the important factor is awareness. With injections and implants and whatever you take, those are things and strategies that would be easier to target to people who are actually aware of and are on PrEP. Now, we are talking and focusing on ethnic minorities and international students. For them, the fact that PrEP in itself, it's such a missing part of their vocabulary, so we still have to go back to the basic and reach out to people. I think it's important to reach out [and provide] basic information.”*

*(PA013, 27 y.o., gay, cis man, India, 4 years in Australia, international student, daily oral PrEP)*

Among participants who were not on PrEP at the time of the interview, many of them reemphasized the need to make PrEP affordable for people without Medicare. As expressed by one participant:

“*If they really want to eradicate and lessen the number of HIV [infections], in their advocacy, making PrEP accessible and much more affordable for people [without Medicare].”*

*(PA017, 32 y.o., same-sex attracted, non-binary, the Philippines, 2.5 years in Australia, international student, not on PrEP with a history of taking PrEP)*

Indeed, several of these participants said that they would consider taking PrEP if it is free for people without Medicare:

“*For students, we don't have much money, so we are very conscious of the financial side of [PrEP], so free [PrEP] is quite important for me, it's a key factor. Make it free.”*

*(PA006, 29 y.o., gay, cis man, Malaysia, 2 years in Australia, international student, never on PrEP)*

## Discussion

This study found that participants' knowledge of and attitudes toward PrEP in Australia have been influenced by the complex intersections between systemic and socio-cultural factors. On a systemic level, Australian health policy does not subsidize PrEP for people without Medicare. As reflected by many participants, the high cost of PrEP without Medicare is a primary barrier to PrEP, especially for international students with limited work rights and relatively low income. This further intersects with the general lack of information about PrEP and access to PrEP without Medicare specific to this population. For many participants, these systemic barriers further intersect with internalized stigma stemming from homo- and sex-negativity both in their country of origin and in Australia, preventing individuals from accessing PrEP (1, 14, 44).

Furthermore, the framing of PrEP as an HIV prevention strategy for at-risk populations, including GBMSM with multiple, casual sex partners with infrequent condom use (45), may further contribute to the stigma around PrEP. This could contradict the cultural expectations of monogamy that adds another layer of complexity (16). For a few participants, concerns over the side-effects of PrEP were influenced by their cultural upbringing around health and medicine, which further intersects with minimum culturally specific information about PrEP that clearly explains the side-effects of PrEP and the benefit of PrEP in preventing acquiring HIV. We recommend that culturally specific information must be developed in collaboration with key stakeholders including HIV organizations, public health professionals, HIV and sexual health specialist and general practice doctors and nurses, as well as members of overseas-born, newly arrived GBMSM communities to address systemic and socio-cultural factors that may prevent people from accessing PrEP, including information about affordable ways to purchase PrEP without Medicare. For these resources to be effective, information provided must be relevant to their lived experiences, ethnicity, and culture (46). As reflected in a study on PrEP uptake among LatinX sexual minority men in the United States, culturally specific information that combines queer and Latin cultures could facilitate an increase in HIV awareness and PrEP uptake among this population (47).

Despite these intersecting barriers, participants who were on PrEP or had a history of taking PrEP reflected on the role of their peers, romantic and/or sexual partner(s) to provide them with information about PrEP, especially accessing and purchasing PrEP without Medicare. Several studies have noted social connectedness as a facilitator to PrEP in Australia (2, 28, 48, 49). We add that health professionals from both private and publicly-funded sexual health clinics play an important role in creating a safe environment for this population to talk openly about sex and sexual health, especially for individuals from strong homo- and sex-negative societies. We also stress the need to equip peers with clear information about PrEP and access to PrEP without Medicare that they can disseminate among their social networks. Providing secure funding to train and support peers in multiple sectors, including in education and health, could increase PrEP knowledge among this population. Additionally, increasing funding for free sexual health clinics to expand their service to include multilingual health services can reduce cultural barriers and further strengthen PrEP awareness and uptake among this population.

Concerning new PrEP modalities, most participants were in favor of injectable PrEP but not PrEP implants, which resembles findings from a previous Australian study (29). However, for this population, the cost of unsubsidized PrEP and limited information about PrEP relevant to their cultures and lived experiences remain an issue that needs to be addressed before new PrEP modalities become available in Australia. This indicates the need for ongoing advocacy to make PrEP free for all people irrespective of their Medicare eligibility so Australia can reach its target of virtual elimination of HIV by 2025 (19, 50, 51). This is to coincide with the development of culturally specific resources to be disseminated in multiple settings including universities, accommodation services, community organizations, and health services (52) to maximize coverage and impact.

Despite our best efforts, the main limitation was that we could not establish relationships and conversations with other ethnic communities in Australia, including the African diaspora communities and the Pacifika communities. The closure of international borders during the COVID-19 pandemic added another challenge in the recruitment process. We were only able to interview participants already in Australia before the international border closure in March 2020, which prevented people from coming to Australia. Furthermore, most participants in this study were international students, and this could impact their health literacy and English language skills that may not reflect the diverse socio-economic, education, health knowledge, and English language proficiency that exist within this population. We were also unable to speak to trans men, and we believe that overseas-born, newly arrived trans men who have sex with men would encounter some unique challenges in understanding and accessing PrEP in Australia, as most PrEP strategies have been directed toward cis men (41). Despite these limitations, this study offers a snapshot of how PrEP has been viewed and understood by this population.

In this study, we found intersections between systemic and socio-cultural factors that influenced participants' knowledge of and attitudes toward PrEP. We believe that a combined strategy of advocacy to make PrEP free for all, culturally specific resources that can be disseminated in various settings, government-funded peer-to-peer programs, and increased capacity of free sexual health clinics to provide a multilingual PrEP service for Medicare-ineligible individuals can play a part in increasing PrEP awareness and uptake among this population. Members of ethnic communities must be included in the design and delivery of these strategies to ensure that they are effective and relevant to their lived experiences, cultures, and identities. For these strategies to be effective, various sectors including and not limited to the government, health, education, accommodation services, and the wider LGBTIQA+ communities must work together to increase PrEP uptake among this population as an integral part of our collective effort of ending HIV transmission in Australia.

## Data availability statement

The datasets presented in this article are not readily available due to the interview transcripts containing participants' personal, sensitive, and identifying data. The Ethics Committee has not approved the publication of this type of data. Interested researchers may contact the Alfred Hospital Ethics Committee if they would like access to the data: research@alfred.org.au, quoting project 84/21.

## Ethics statement

The studies involving human participants were reviewed and approved by the Alfred Hospital Ethics Committee, Project Number 84/21. The patients/participants provided their written informed consent to participate in this study.

## Author contributions

BS, TRP, EPFC, and JJO were involved in the conceptualization and design of the research. BS, TRP, EPFC, JJO, and JA assisted in the recruitment. BS interviewed the participants, analyzed the data, and drafted the manuscript. BS and TRP cross-checked the themes that emerged from the data. TRP, EPFC, and JJO reviewed the data analysis. All authors were involved in the revision of the manuscript.

## Funding

EPFC and JJO are each supported by an Australian National Health and Medical Research Council (NHMRC) Emerging Leadership Investigator Grant (GNT1172873 for EPFC and GNT1193955 for JJO). CKF was supported by an NHMRC Leadership Investigator Grant (GNT1172900). NM was supported by an NHMRC Early Career Fellowship (GNT1158035).

## Conflict of interest

The authors declare that the research was conducted in the absence of any commercial or financial relationships that could be construed as a potential conflict of interest.

## Publisher's note

All claims expressed in this article are solely those of the authors and do not necessarily represent those of their affiliated organizations, or those of the publisher, the editors and the reviewers. Any product that may be evaluated in this article, or claim that may be made by its manufacturer, is not guaranteed or endorsed by the publisher.
