# Supplementary material for: “How PrEPared are you?”: Knowledge of and attitudes toward PrEP among overseas-born and newly arrived gay, bisexual, and other men who have sex with men in Australia
Source: Front Public Health. 2022 Aug 19;10:946771. doi: 10.3389/fpubh.2022.946771 (PMC9437584; doi:10.3389/fpubh.2022.946771)
Supplement: Supplementary file 1 [file Table_1.pdf]

frontiers

Supplementary Material

Supplementary Table 1. Place of recruitment and recruitment strategy

LGBTIQ+ community organization	Recruitment strategy
Australian GLBTIQ+ Multicultural Council (AGMC) – a national multicultural/multifaith LGBTIQ+ organization.	Post on Facebook group.
LatinX and Hispanic Rainbow Community (LAHRC) – a community organization for LatinX LGBTIQ+ in Australia	Post on Facebook group
Queer Refugee and Asylum Seeker Peers (QRASP) – a not-for-profit organization for LGBTIQ+ asylum seekers and refugees in Victoria, Australia	Recruitment email to members.<br>Posters at the office.<br>Word of mouth.
Melbourne Glamourhead Sharks (GLAMS) – a swimming club for the LGBTIQ+ community in Victoria, Australia.	Recruitment email and digital flyer to members.
PrEP Access Now (PAN) – a community-led organization to provide information about PrEP to gay and bisexual men in Australia.	Post on Facebook group.
PrEP'd for Change – an online community for gay and bisexual men that provide information about PrEP.	Post on Facebook group.
Thorne Harbour Health – an LGBTIQ+ community health organization in Victoria, Australia	Recruitment email and digital flyer to members
Non-LGBTIQ+ community organization	
Council of International Students Australia (CISA) – a not-for-profit, student-lead organization for international students in Australia	Recruitment email and digital flyer to members.
Story Is Connection – a not-for-profit arts organization for international students in Victoria, Australia	Recruitment email and digital flyer to members.
Health clinics and organization	
Melbourne Sexual Health Centre (MSHC) – the largest, publicly funded HIV/STI clinic in Victoria, Australia	Post on MSHC Twitter account.<br>Recruitment flyer provided to clients by medical practitioners.
Alfred Health PrEPMe Clinic – a publicly funded, nurse-led PrEP clinic that provides a specialized service for people without Medicare.	Recruitment flyer provided to medical practitioners to promote the study to their clients.

Monash University Health Services	Recruitment flyers provided to staff and practitioners to promote the study to their clients.
University	
Melbourne University student portal	Recruitment post.
Monash University Central Clinical School research portal	Recruitment post.
Professional Network	
HIV/STI CALD and Overseas born gay men network	Recruitment email and digital flyer to members.
International Student Sexual Health Network	Recruitment email and digital flyer to members.

Supplementary Table 2. Information and questions about new PrEP Modalities

Information	Question
Injectable PrEP	
"At the moment, there is a trial on injectable PrEP. This means that you will only need to receive an injection of PrEP once every two months. You don't need to take a PrEP pill every day."	"Is this something that you might be interested in if it becomes available in Australia?"<br>"Can you please tell me why you might be interested/not interested in injectable PrEP?"
PreP implants	
"There is also another study to investigate PrEP implants. This means that they will insert an implant containing PrEP under the skin of your arm. The study suggests that you will only need to replace the implant once or twice a year. You don't need to take a PrEP pill every day."	"Is this something that you might be interested in if it becomes available in Australia?"<br>"Can you please tell me why you might be interested/not interested in PrEP implants?"
